# Semiology Extraction and Machine Learning–Based Classification of Electronic Health Records for Patients With Epilepsy: Retrospective Analysis

**DOI:** 10.2196/57727

**Published:** 2024-10-17

**Authors:** Yilin Xia, Mengqiao He, Sijia Basang, Leihao Sha, Zijie Huang, Ling Jin, Yifei Duan, Yusha Tang, Hua Li, Wanlin Lai, Lei Chen

**Affiliations:** 1 Department of Neurology, West China Hospital, Sichuan University, #37 Guoxue Alley, Wuhou District, Chengdu, China, 86 18980605819; 2 Sichuan Provincial Engineering Research Center of Brain-Machine Interface, and Sichuan Provincial Engineering Research Center of Neuromodulation, West China Hospital, Sichuan University, Chengdu, China

**Keywords:** epilepsy, natural language processing, machine learning, electronic health record, unstructured text, semiology, health records, retrospective analysis, diagnosis, treatment, decision support tools, symptom, ontology, China, Chinese, seizure

## Abstract

**Background:**

Obtaining and describing semiology efficiently and classifying seizure types correctly are crucial for the diagnosis and treatment of epilepsy. Nevertheless, there exists an inadequacy in related informatics resources and decision support tools.

**Objective:**

We developed a symptom entity extraction tool and an epilepsy semiology ontology (ESO) and used machine learning to achieve an automated binary classification of epilepsy in this study.

**Methods:**

Using present history data of electronic health records from the Southwest Epilepsy Center in China, we constructed an ESO and a symptom-entity extraction tool to extract seizure duration, seizure symptoms, and seizure frequency from the unstructured text by combining manual annotation with natural language processing techniques. In addition, we achieved automatic classification of patients in the study cohort with high accuracy based on the extracted seizure feature data using multiple machine learning methods.

**Results:**

Data included present history from 10,925 cases between 2010 and 2020. Six annotators labeled a total of 2500 texts to obtain 5844 words of semiology and construct an ESO with 702 terms. Based on the ontology, the extraction tool achieved an accuracy rate of 85% in symptom extraction. Furthermore, we trained a stacking ensemble learning model combining XGBoost and random forest with an *F*_1_-score of 75.03%. The random forest model had the highest area under the curve (0.985).

**Conclusions:**

This work demonstrated the feasibility of natural language processing–assisted structural extraction of epilepsy medical record texts and downstream tasks, providing open ontology resources for subsequent related work.

## Introduction

Epilepsy is a major chronic neurological disorder that affects approximately 70 million people and severely reduces the quality of life of patients and their families [1]. Obtaining a correct and complete seizure semiology efficiently is essential for the diagnosis and classification of seizures. However, this process is difficult to achieve. First, the symptoms of seizures are stereotypical but variable, and the same seizure course is in fact a complex combination of multiple symptomatologic elements in time and space. Furthermore, the type of seizure an individual patient experiences can change over the course of the disease [2,3]. Second, seizures have sudden onset, resulting in a short period of time for patients or witnesses to recognize and observe them, and history taking often relies on experienced and careful questioning by epilepsy specialists rather than recording the patient’s statements directly [4,5]. Finally, epilepsy specialists are scarce and unevenly distributed worldwide. Nonneurologists, medical students, caregivers, and community workers play important roles in epilepsy care but lack appropriate tools to tease out epilepsy histories and determine classifications [6-9].

In recent years, natural language processing (NLP) has been widely used in the structured processing of clinical text data and development of intelligent diagnostic tools in neurology [10]. NLP methods have been used to automatically extract details from electronic health records (EHRs) of patients with epilepsy, such as categorical diagnosis, abnormal electroencephalogram (EEG) and imaging results, and medications prescribed [11-13]. These data are also used to accomplish tasks such as automated identification of cohorts of drug-resistant patients and long-term prognostic tracking [14,15]. However, the complexity of epilepsy symptom elements remains a challenge for entity recognition and automatic extraction classification.

Therefore, ontologies were introduced to address this complexity. The concept of ontology is derived from philosophy and is used for formal, structured, domain-specific, and human- and computer-interpretable representations of entities and relationships. It has been widely used in computers, bioinformatics, and medical informatics [16,17]. Application ontology can be used in the medical field to represent established knowledge within a domain and maintain a standardized vocabulary across multiple locations, datasets, and consortiums, allowing for automated computation and decision-making based on structured data. Application ontologies can also be combined with NLP techniques to disambiguate textual concepts and build tools for the knowledge extracted from EHRs [10,18]. This work demonstrated the feasibility of NLP-assisted structural extraction of epilepsy medical record texts and downstream tasks, providing open ontology resources for subsequent related work.

## Methods

### Dataset

Electronic medical record data were obtained from patients with an *International Classification of Diseases, Tenth Revision* (*ICD-10*) epilepsy diagnosis (G40 or G40.x) who were hospitalized at West China Hospital of Sichuan University and assigned an epilepsy diagnosis between 2010 and 2020. The seizure type of inpatients was determined by discharge diagnosis.

The text information of the current medical history records the details of the occurrence, evolution, diagnosis, and treatment of the patient’s disease; is written in chronological order; and is divided into the following parts: onset of the disease, including the time and place of onset; antecedent symptoms; probable causes or triggers; characteristics of the main symptoms and their development and change (describing the location, nature, duration, degree, factors of relief or aggravation, and evolution of the main symptoms in sequential order); accompanying symptoms; diagnosis and treatment since the onset of the disease; and the patient’s general condition since the onset of the disease.

### Ethical Considerations

The study was reviewed and approved by the Ethics Committee of West China Hospital of Sichuan University (2022(1083)). Since the data were obtained from previous medical records, we have received approval from the ethics committee for a waiver of informed consent. The study data were deidentified, and the privacy and personal information of the subjects were protected.

### Framework for Standardizing Seizure Information

**Figure 1.**
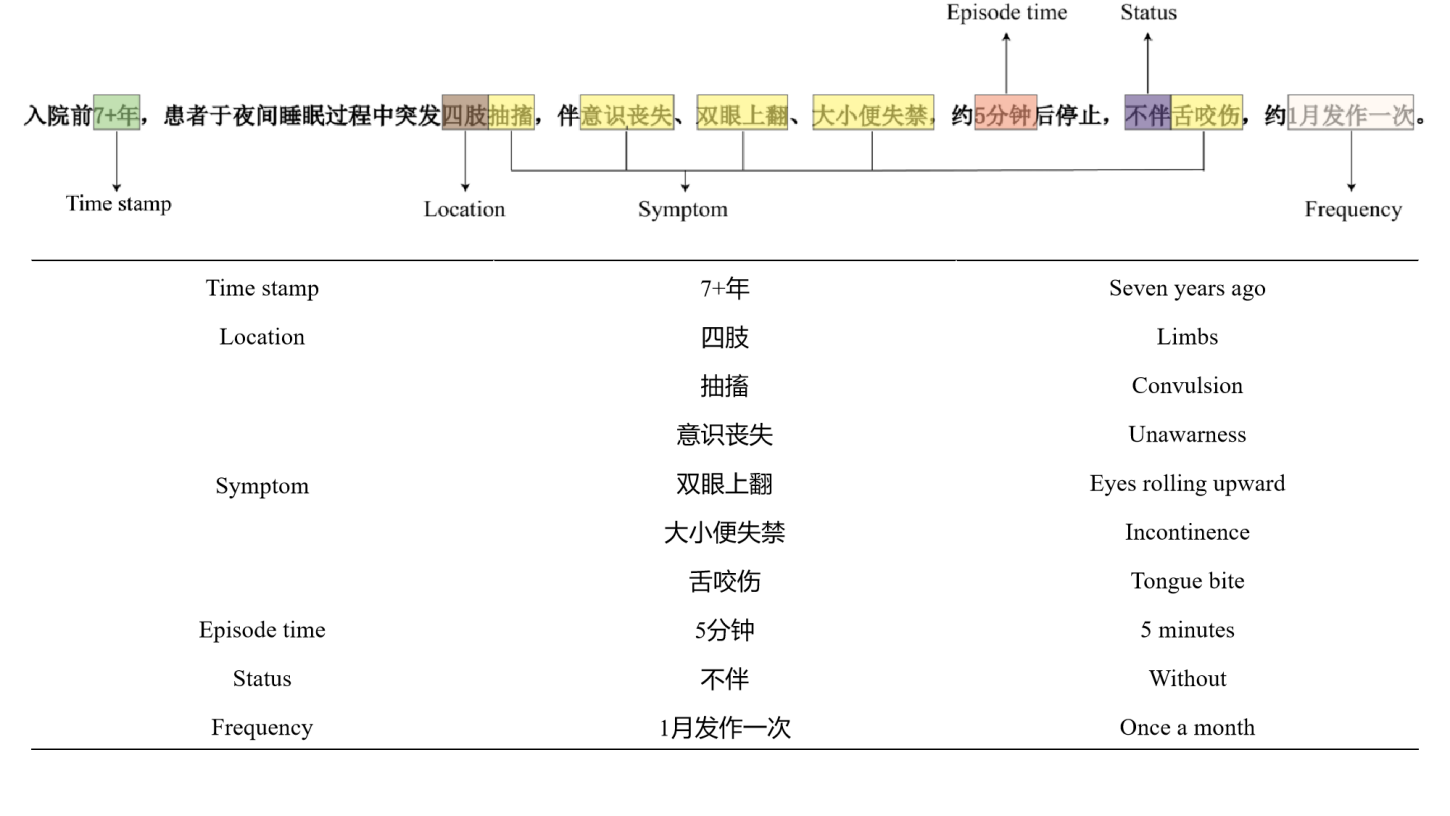
Example of the standardized framework.

We proposed a seizure extraction framework for mining and structuring important information related to seizures from the presenting medical histories of patients with epilepsy (Figure 1). The framework requires the extraction of the following information:

1. Time stamp: The important point in time at which the patient’s condition has changed since today.
2. Location: Seizure site refers to the anatomical parts of the body corresponding to the symptom performance.
3. Symptom: Symptom performance refers to the symptoms and signs that appear during the seizure.
4. Duration of seizure event (episode time): Duration of epileptic events within the seizure episode.
5. Status: Occurrence state refers to the state corresponding to the symptom performance, including “with,” “without,” or “unknown.”
6. Frequency: The frequency of seizures, for example: once a month, and so forth.

### Labeling Process

Six annotators completed the labeling process. Four of them, junior physicians (SB, LS, LJ, and YD) specializing in epilepsy or epilepsy researchers, were responsible for independently extracting seizure-related information from 2500 raw texts of presenting medical histories according to a standardized framework. Two senior physicians (HL and WL) specializing in epilepsy were responsible for discussing and formulating the framework of the annotation and the rules that should be followed during annotation to ensure reliability, providing uniform training to the annotators, and manually reviewing the final results of the annotation. Annotation rules included the following:

When a particular Chinese phrase used to describe the seizure process was a fixed collocation, the phrase was extracted as a whole without separating the verb and the object (usually a location) in it individually, in order to avoid a decrease in the specificity of the extraction.

Due to the specificity of the commonly used symptomatology phrases in the Chinese section, it is important to ensure that the symptomatic manifestations are extracted at the coarsest possible granularity, that is, descriptive phrases that include seizure state and seizure site are avoided. However, phrases should not be disassembled when they cannot be clearly recognized as symptoms, such as lip smacking (oropharyngeal automatisms) and hand rubbing (hand automatisms), and the anatomical part of the phrase should be retained. It should also be confirmed that all seizure symptomatology is extracted from seizures and not from other symptoms accompanying epilepsy. Cognitive decline, such as memory and attention, should not be included in labeling.

Do not standardize the presentation of the extracted information and keep it as original as possible.

To assess the consistency of the annotations by the 4 annotators, 50 identical medical records were included without their knowledge. Two senior physicians provided reference standards for the annotation of the 50 medical records. We used Fleiss’s κ to calculate interannotator agreement. By convention, κ value above 0.80 indicates “near-perfect” agreement.

### Bilingual Ontology Construction for Seizure Semiology

Compared with other parts of the seizure information framework, epileptic semiology expression and the diversity of expression extraction tasks are more challenging, especially for Chinese EHRs of epilepsy. Therefore, we constructed a bilingual ontology to share the lexicon obtained from manual extraction and annotation. It can be further used, evaluated, and refined for future Chinese epilepsy history extraction tasks.

We defined the scope of this domain of ontology as epileptic semiology by reference, reused the more authoritative epilepsy-related ontologies and terminology sets as standard terminology, referred to the basic formalized ontology (BFO) as the top-level ontology, and hierarchically arranged the entities according to their domain-neutral framework. Then, we deemphasized the annotated symptoms collected in the annotation phase to eliminate redundancy and placed them into the corresponding terms as their synonymous expression properties. We used Protégé as the editor of the ontology and uploaded it in Ontology Web Language (OWL) as the first version of the world’s largest ontology browser, BioPortal.

### Extraction Process and Evaluation of Extraction Results

We used some NLP tools to structure the extraction of current medical history from EHRs. We imported the organized dictionaries of symptom performance, symptom nature, seizure frequency, and seizure site into the Jieba tokenizer and initialized the Part-of-Speech Tagger (Postagger) and Dependency Parser (Parser) of the pyltp [19] plug-in using existing models (pos.model, parser.model). pyltp provides a series of Chinese NLP tools, and users can use these tools for Chinese text segmentation, part-of-speech tagging, parsing, and so on.

**Figure 2.**
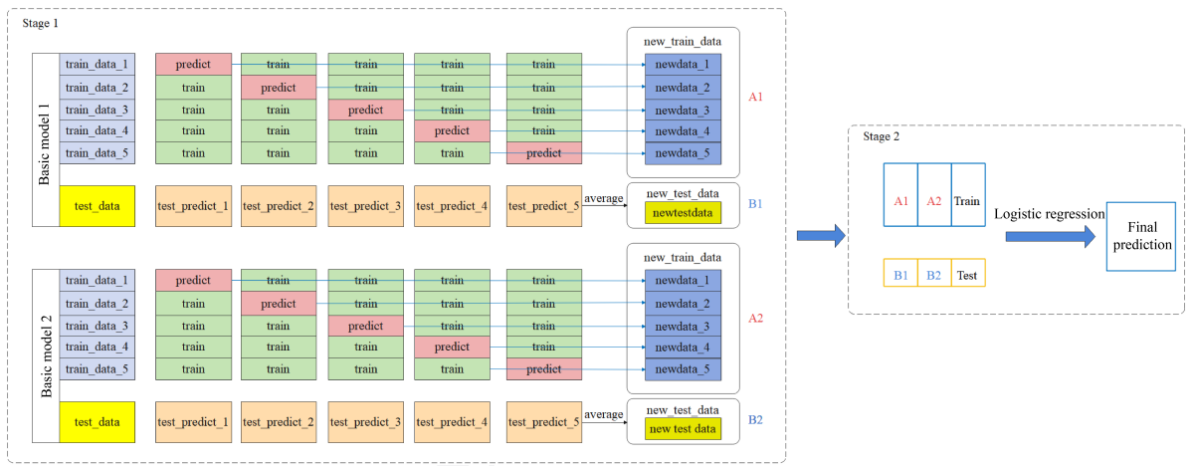
Extraction modeling workflow.

Specifically, in the data preprocessing stage, we first imported organized dictionaries of symptom presentation, symptom type, seizure frequency, and seizure location. These dictionaries are used for subsequent segmentation and feature extraction. We used Postagger to tag the parts of speech of the tokenized results and Parser to analyze the dependency relations of the words in the current sentence or context. Next, we performed text segmentation and annotation, using Jieba Segmenter to segment the medical history text in the EHR. Jieba Segmenter is able to accurately slice and dice the text based on the imported dictionaries. Postagger was called to lexically annotate the segmentation results by identifying the lexical properties of each word. The dependencies between words are analyzed using Parser to determine the syntactic structure between words. Then, to extract symptom information, we iteratively processed the participle results by combining a list of negatives, a list of transitive or logical connectives, and a list of temporal adverbs. These normalized lists allowed us to accurately identify positive and negative symptom information. In each sentence, information such as the location, type, duration, and frequency of symptom episodes was extracted. Finally, the extracted information such as positive and negative symptoms, location, nature, duration, and frequency of episodes was structured and stored in the output dictionary according to the temporal nodes. The overall process flow is illustrated in Figure 2.

The software and programming languages used included Python 3.8.8, pyltp 0.2.0, pandas 1.4.2, and Jieba 0.42.1.

After the extraction was completed, we randomly selected 200 cases from all the results for manual inspection to comprehensively assess the extraction capability and obtain the accuracy for 6 aspects separately: time stamp, symptom, location, episode time, status, and frequency.

### Seizure Classification Based on Machine Learning

Our work aimed to build a binary classification model capable of distinguishing between generalized and focal seizures. The analysis process, based on supervised machine learning, consisted of the following steps: data preprocessing, feature selection, algorithm selection, parameter tuning, and performance evaluation.

### Data Preprocessing

Our extraction tool was used to retrieve semiology data of the patients. After preprocessing 16,587 records by *ICD* coding combined with regular expression matching, 10,098 records were excluded because they did not receive a clear classification (60%).

A total of 6489 medical history text records with a diagnosis of generalized or focal seizure were retained, including 2632 records of generalized epilepsy and 3857 records of focal epilepsy. After communication with clinicians, 103 symptom words were defined to cover the main symptoms that can occur in patients with epilepsy. We used text-matching techniques to map the symptom descriptions in each record to these 103 symptom words. Specifically, for each record, if a symptom word was mentioned in the text, we marked the corresponding symptom word as 1; if it was not mentioned, it was marked as 0. For example, if a record mentioned “Clonic” but not “Foaming at Mouth,” then the field for “Clonic” was set to 1, and the field for “Foaming at Mouth” was set to 0.

### Feature Selection

We used several feature selection techniques to identify the most relevant features for the classification task. Specifically, we used recursive feature elimination, random forest–based feature importance, mutual information, and the SelectKBest method using the ANOVA *F* value. Each method was systematically applied to the feature matrix (X) and the label vector (y) to generate a reduced set of features. We varied the number of retained features (k) across multiple values to evaluate its impact on model performance. In addition, we examined the effects of different sample ratios on the model’s performance.

### Algorithm Selection and Parameter Tuning

**Figure 3.**
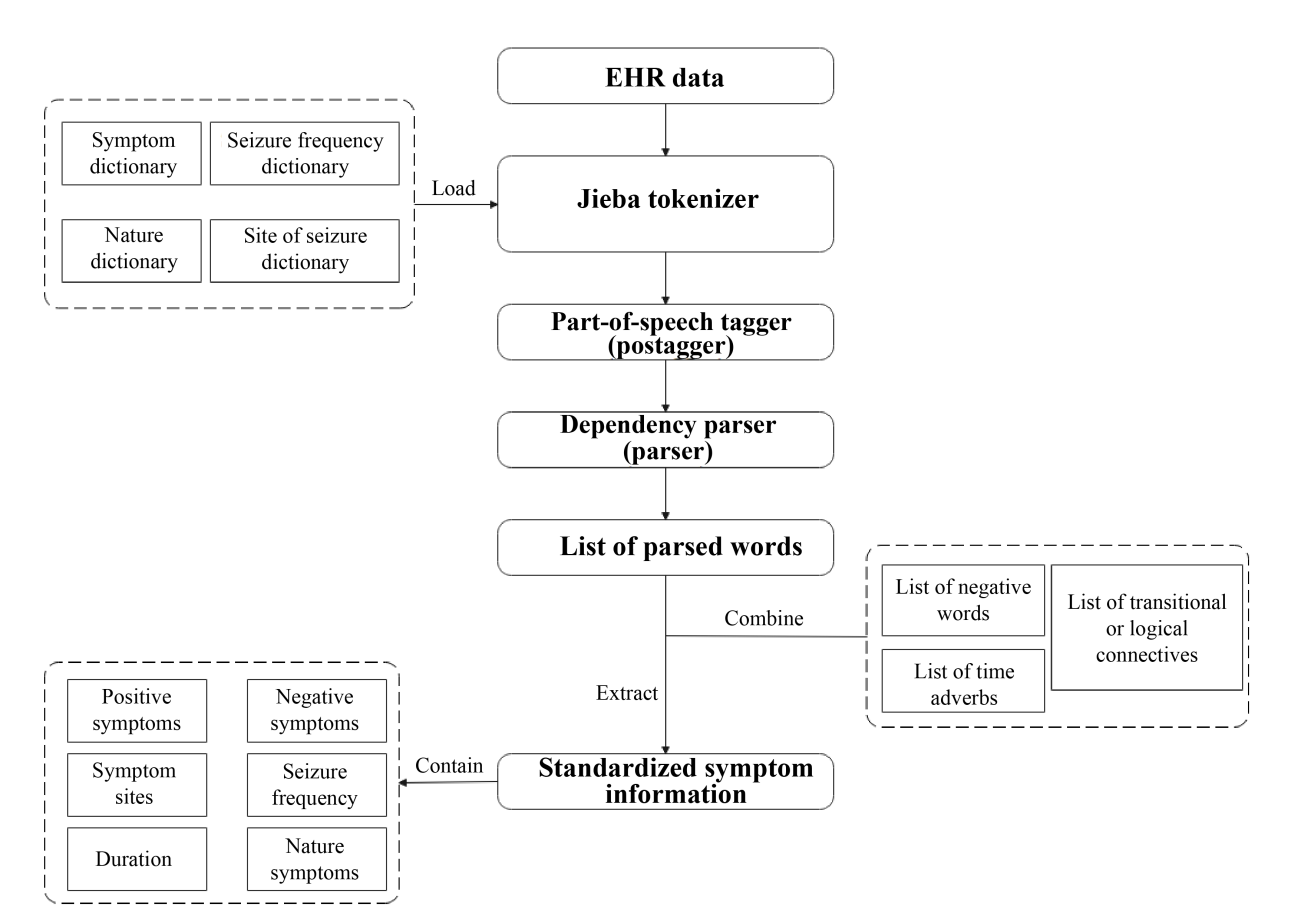
Stacking integration learning process. EHR: electronic health record.

Subsequently, we divided the preprocessed dataset into training and testing sets at a 7:3 ratio. We used 4 types of models as base models: decision tree [20], random forest [21], XGBoost [22], and LightGBM [23]. Using grid search algorithms and k-fold cross-validation, we optimized the hyperparameters of the models with training to enhance the model accuracy. Specific parameters are detailed in Multimedia Appendix 1. We also introduced the stacking ensemble learning method, which was conducted in 2 stages, as illustrated in Figure 3. In the first stage, we performed 5-fold cross-validation. Specifically, we divided the training dataset into 5 parts, with 4 serving as the training set for base model training and the remaining part serving as the validation set for generating new training data. Simultaneously, we predicted the entire test set (test_data) to create a new test dataset. In the second stage, we used the training and testing sets generated from the first stage as inputs for further training and prediction using the logistic regression model, resulting in the final outcome. In this study, we combined the XGBoost model with the random forest and LightGBM models for combined training and testing.

### Performance Evaluation

Finally, we used the test set to evaluate the precision, recall, *F*_1_-scores, and the area under the receiver operating characteristic curve (ROC) value of the model. We designated “generalized epilepsy” as label A and “focal epilepsy” as label B. TP(*A*) represents true positives, FP(*A*) represents false positives, and FN(*A*) represents false negatives for label A, and similarly for label B.

Precision is defined by the following formula:


(1) $$Precision=\frac{1}{2}\left(\frac{TP(A)}{TP(A)+FP(A)}+\frac{TP(B)}{TP(B)+FP(B)}\right)$$

Recall is defined by the following formula:


(2) $$Recall=\frac{1}{2}\left(\frac{TP(A)}{TP(A)+FN(A)}+\frac{TP(B)}{TP(B)+FN(B)}\right)$$

The *F*_1_-score (*F*_1_) is defined by the following formula:


(3) $$F_{1}=\frac{2\times Precision\times Recall}{Precision+Recall}$$

For the classification analysis of seizure, the following software or programming language versions were used: Python 3.8.8, NumPy 1.24.3, pandas 1.4.2, scikit-learn 1.3.2, XGBoost 2.0.1, and LightGBM 4.1.0.

### Bilingual Ontology Construction for Seizure Semiology

Compared with other parts of the seizure information framework, epileptic semiology expression and the diversity of expression extraction tasks are more challenging, especially for Chinese EHRs of epilepsy. Therefore, we constructed a bilingual ontology to share the lexicon obtained from manual extraction and annotation. In developing epilepsy semiology ontology (ESO), we followed 5 of the 7 steps of the Stanford methodology: (1) defining the domain and scope of the ontology, (2) reusing existing ontologies to the extent possible, (3) enumerating ontology terms, (4) defining classes and class hierarchies, and (5) defining class attributes (Multimedia Appendix 2).

In the first step, epileptologists and the ontology development team met biweekly to define the scope of the ontology and to ensure that the goals remained constant throughout its development. In steps 2 and 3, we standardized terminology by referring to existing, more authoritative epilepsy-related ontologies and terminology sets. In the fourth step, we adopted the BFO as the top-level ontology. In the fifth step, we de-emphasized the annotated symptoms collected in the annotation phase to eliminate redundancy and placed them into the corresponding terms as their synonymous expression properties. Finally, we rendered the ontology using the OWL in the Protégé ontology editor and uploaded it to the world’s largest ontology browser, Bioportal, as a first version.

## Results

### Patient Cohort

The study cohort included 10,925 patients and 10,658 texts of presenting medical histories. The patient cohort included 42% (4588/10,925) females and 58% (6337/10,925) males with a mean age of 31.45 (age range: 1‐92) years. The presenting medical history texts were independently written and completed by 117 physicians. Fifty-seven percent (6227/10,925) of the patients in the patient cohort ultimately received a definitive diagnostic classification of seizures at the time of discharge, with 32% (1992/6227) of patients having focal epilepsy and 26% (1619/6227) having generalized epilepsy.

### Assessment of Labeling Quality Control Results and Extraction Capacity

In the annotation phase, we assigned 50 identical texts to the annotators without their knowledge to test the consistency of their annotations. The κ-value of the 4 annotators was 0.862, indicating a high degree of consistency.

After completing the extraction using the model, we manually inspected a random sample of 200 notes from the extraction results (which included 235 seizures) to assess the extraction performance of the model. The extraction results for the 5 dimensions are shown in Table 1.

**Table 1.** Extraction performance.

	Time stamp	Location	Symptom	Episode time	Status	Frequency
Total number of elements by reviewer annotation, “gold standard”	235	512	1325	183	1325	106
Total number of elements by algorithm report	196	516	1219	175	1302	93
Number of correct algorithm-reported elements	181	507	1126	145	1254	84
Recall, n/N (%)	181/235 (77)	507/512 (99)	1126/1325 (85)	145/183 (79)	1254/1325 (95)	84/106 (79)
Precision, n/N (%)	181/196 (92)	507/512 (98)	1126/1219 (92)	145/175 (82)	1254/1302 (96)	84/93 (90)
*F*_1_-score	0.83	0.98	0.88	0.80	0.95	0.84

### Epilepsy Semiology Ontology

The overall hierarchical structure of ESO adheres to the architecture of the top-level ontology BFO, which supports semantic interoperability between ontologies, starting from “continuant” and “occurrent” under “entity.”

The ESO contains a total of 176 terms, most of which are based on the nominal entity “anatomical entity” and the process “physiological pathological process,” with a maximum depth of 10 layers. According to the principle of ontology reuse, we partially reused and rearranged the concepts of “pathophysiological process” and its leaf nodes in epilepsy and seizure ontology (EPSO) [24] and also referred to the existing semiology terminology collection of the International League Against Epilepsy, which includes a total of 132 epilepsy semiology terms. In terms of seizure sites, we referred to the “Bodily Feature” section of Systemized Nomenclature of Medicine Clinical Terms (SNOMED CT) [25] and EPSO, which contains a total of 32 seizure-site terms. The purpose, scope, language, and users are listed in Multimedia Appendix 3.

As an important step in implementing the medical record extraction function of the application ontology, we added Chinese translations and synonyms of symptom performance as entity attributes (Multimedia Appendix 3). After annotating 2500 medical records, we obtained 5844 words of semiology. After de-emphasizing and removing nonepileptic seizure symptoms (usually abnormal general conditions and comorbid symptoms), we obtained 702 terms, 75 primary terms, and their synonyms. Among them, there were more than 30 synonyms for holding, dropping, and vocalization.

### Performance of Seizure Classification

**Figure 4.**
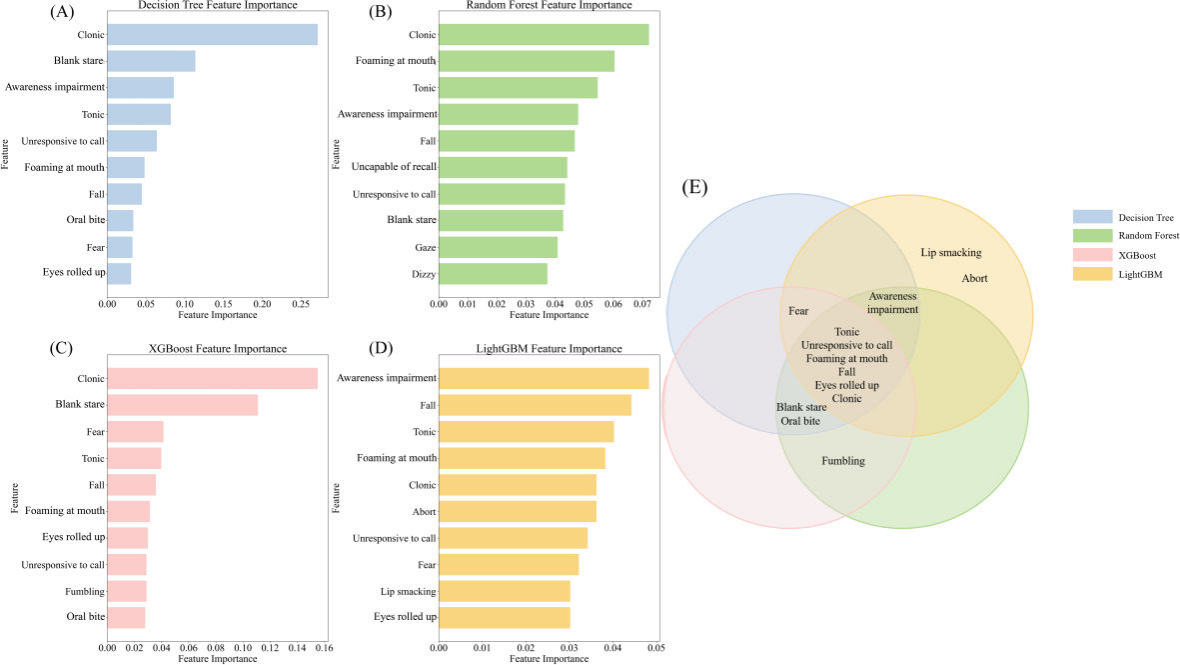
Distribution of important features of the base model. (A) Decision tree model important features. (B) Random forest model important features. (C) XGBoost model important features. (D) LightGBM model important features. (E) important features of the base model Wayne chart.

In the feature selection process, we found that choosing 103 features among the 4 feature selection methods gave the best results, and we also observed that choosing different sample ratios for training had little impact on the model performance (Multimedia Appendix 4). On this basis, we optimized the parameters and trained 4 foundational models—decision tree, random forest, XGBoost, and LightGBM—to distinguish between generalized and focal epilepsy. Figure 4A-E illustrates the contribution of each symptom feature to the predictive decisions of these models. Notably, “clonic,” “tonic,” “unresponsive to call,” “eyes rolled up,” “foaming at mouth,” and “fall” are pivotal in differentiating seizure types.

**Figure 5.**
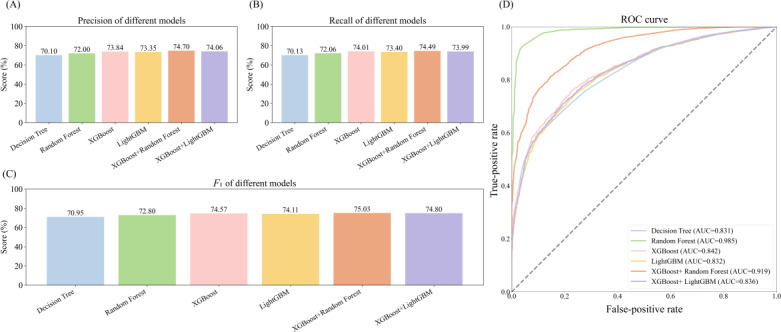
Comparison of model evaluations plotted against ROCs. (A) Comparison of precision across models. (B) Comparison of recall across models. (C) Comparison of *F*_1_-scores across models. (D) Comparison of ROCs across models. AUC: area under the curve; ROC: receiver operating characteristic curve.

In addition, we trained a stacking ensemble learning model. As shown in Figure 5A-C, the stacking ensemble model outperformed the other base models in terms of precision, recall, and *F*_1_-score. Among them, the ensemble model combining XGBoost and random forest yielded the best results, with the highest *F*_1_-score (75.03%). We also compared the ROCs of the various models represented by different colors. Notably, the random forest model and XGBoost+random forest ensemble model outperformed the other models, as indicated by the orange and blue lines, respectively. As shown in Figure 5D, the random forest model had the highest area under the curve (AUC)—0.984—whereas the XGBoost+random forest ensemble model had an AUC of 0.919, with the AUCs of the other models falling below these 2.

**Figure 6.**
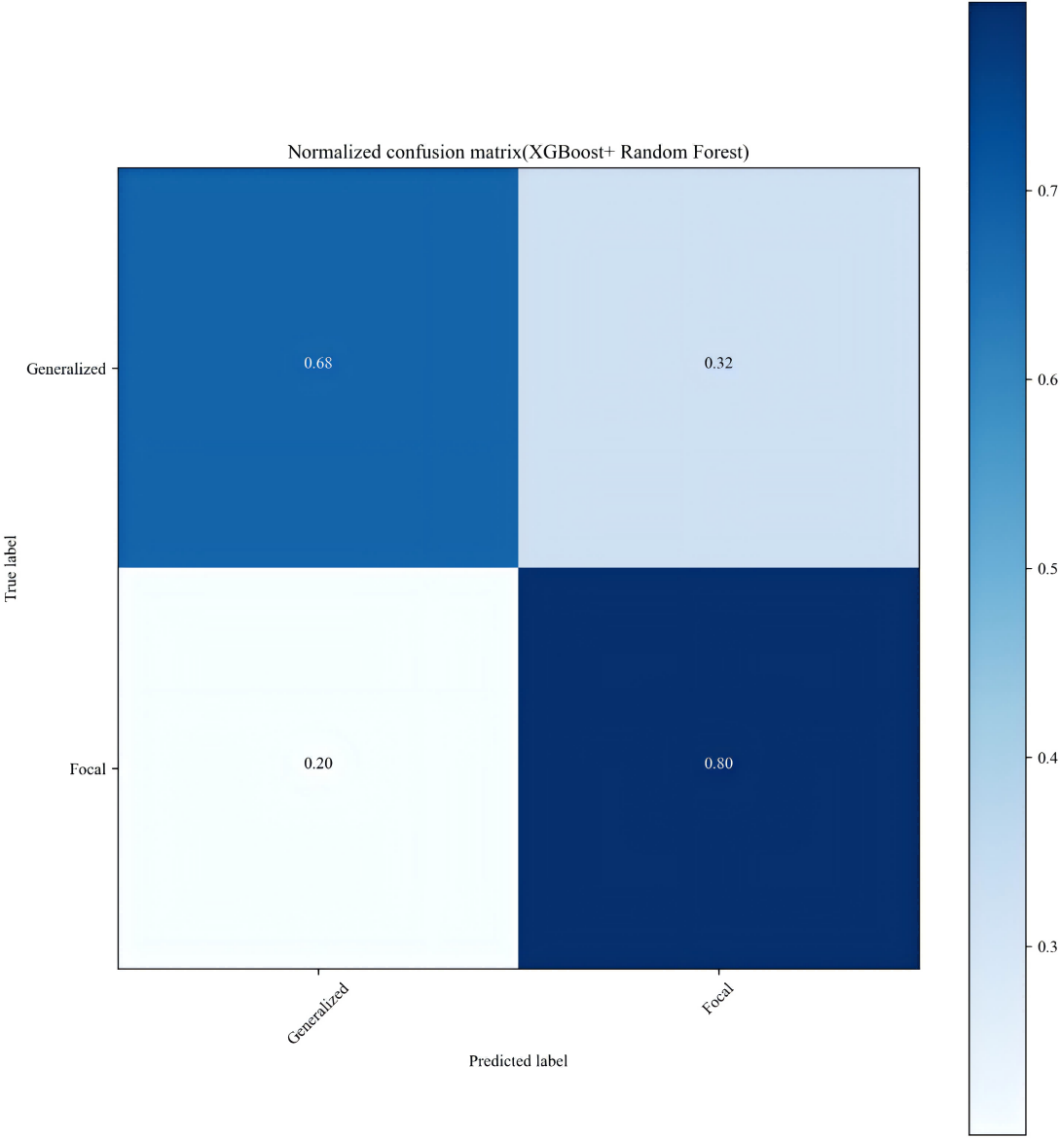
XGBoost+random forest confusion matrix plot.

Ultimately, we selected the ensemble model combining XGBoost and random forest for predicting seizure classification and visualized its confusion matrix. As shown in Figure 6, the model has a precision of 0.68 for predicting “generalized epilepsy” and a precision of 0.80 for predicting “focal epilepsy.”

## Discussion

### Principal Findings

In this study, the first Chinese-English ontology of epilepsy semiology was established, the first non–English-structured extraction of epilepsy history text was achieved by combining manual annotation and NLP techniques, and automatic seizure classification was further accomplished based on the data extracted by the tool.

### Comparison to Prior Work

Ninety percent of the disease burden caused by epilepsy is borne by resource-limited countries. China has more than 12% of patients with epilepsy worldwide [26,27]. The Global Burden of Disease study reported that, in 2019, China’s disability-adjusted life years (DALYs) due to epilepsy accounted for 10% of the global DALYs and 94% of the DALYs in East Asia [28]. However, the development of Chinese language EHR processing tools for epilepsy has been delayed because of the lack of high-quality corpora such as relevant terminology sets. English ontologies and terminology systems, including SNOMED CT, Unified Medical Language System, and EPSO [26], are limited by the problems of diverse descriptions of Chinese medical entities, fuzzy boundaries, and the existence of nested relationships. Therefore, it is more difficult to support clinical terminology extraction from Chinese medical records after “Chinese-ization” [29]. The technical challenges of Chinese NLP lie in its complex word-splitting process, high-frequency ambiguity phenomenon, and flexible and variable sentence construction [30]. By contrast, English NLP is relatively simple to process because of its clear separation of words by spaces, more standardized syntactic structures, and abundant processing resources. Despite these differences, the gap between Chinese and English NLP technologies is gradually narrowing as deep learning and pretrained language models continue to advance and multilingual processing capabilities are significantly enhanced. In this study, the ontology and extraction tool constructed based on the corpus of the Southwest Epilepsy Center can better serve the grassroots areas in western China, where the burden of epilepsy is high and medical resources are relatively scarce, thereby bridging the world’s health disparities for people with epilepsy [26,31].

In this study, for the first time, the symptom elements of epileptic seizures were extracted at an ultrafine granularity, the accuracy of the extraction of the features reached 0.85, and the classification of generalized and focal seizures relying on the symptom features alone reached an AUC of 0.985. We also found that the key features in the classifier corresponded to the “red flag” symptoms used by human experts, yielding a list of symptoms including “clonic,” “tonic,” “unresponsive to call,” “eyes rolled up,” “foaming at mouth” and “fall,” which are the same basic key features as those categorized by human experts’ guidelines [2]. To the best of our knowledge, this is the first time that a present history of epilepsy has been extracted and automatically categorized with symptom element granularity [32,33]. Barbour et al [34] created regular expressions manually as well as creating false-positive filters and disambiguated them using conditional matching to extract entities such as seizure type, with internally tested *F*_1_-values ranging from 0.86 to 0.90. Vulpius et al [35] extracted seizure epilepsy types primarily by manually constructing dictionaries.

However, these 2 studies were based only on existing unstructured diagnostic texts rather than indirect inference through medical history texts, and only automated extraction, rather than automated classification based on symptom features, was achieved. In our seizure classification task, we used a stacking integration technique to combine the XGBoost and random forest models (AUC=0.919). Despite the higher AUC of the random forest model, it may have lower precision or recall in some categories, resulting in a less favorable *F*_1_-score than the stacking method. The stacking method, on the other hand, by combining the advantages of both random forest and XGBoost, may achieve a more balanced performance across all categories, thereby improving the *F*_1_-score.

Although downstream tasks for seizure classification currently exist, most rely on a single-model architecture, such as support vector machine, linear model, or XGBoost [35,36]. However, by pooling multiple underlying models using stacking techniques, it is possible to improve model performance and reduce the risk of overfitting, which in turn improves the model’s generalization capabilities.

### Future Directions

Beyond the initial diagnosis and classification of seizure, our study has the potential to identify specific types of epilepsy. For example, the classification of adolescent myoclonic epilepsy may change over the course of a single patient’s illness, with a predominance of absence and myoclonic seizures initially, followed by intensification of generalized tonic-clonic seizures in adulthood or after practice tasks [3]. This type of epilepsy is difficult to recognize because of changes from pediatric and adult neurologists. Plug-ins based on extraction and classification models can be developed to alert epileptologists to consider this particular type.

In addition, accurate extraction of seizure duration and frequency has been used in epilepsy research to help clinical researchers accurately screen retrospective cohorts in vast multicenter electronic health information databases, for example, by accelerating the speed of patient recruitment and data collection, screening of rare epilepsy cohorts [37], and screening of persistent status epilepticus in children [38]. The extracted data also enable the dynamic and automated monitoring of postmedication efficacy, epidemiological statistics, and medical economics studies on a larger scale. In the future, we will consider the use of deep learning models and the addition of multimodal features such as imaging and EEG in the seizure classification task to achieve a more accurate and dynamically changing classification capability based on the patient’s journey. With further improvements in extraction and classification accuracy, automated symptom-based classification will be uniquely suited to help primary care physicians and other specialists accurately classify epilepsy and select appropriate medications. In conclusion, this work demonstrates the feasibility of NLP-assisted structured extraction of epilepsy history text and downstream tasks in Chinese and provides an open ontology resource for subsequent related work.

### Limitations

This study also has some limitations. First, including the fact that the data source was only from a single center, we have not yet verified its transferability to other regions in China. Second, we have not yet applied the ontology to real clinical scenarios, such as assisting clinicians in structured and efficient registration of epilepsy history. Third, the accuracy of dependent syntax analysis is crucial to the effectiveness of information extraction, and the flexibility of Chinese grammar adds to the difficulty of the analysis. Fourth, although current deep learning techniques have gained momentum to improve the situation, they also require finer tuning and extensive contextual adaptation testing. Fifth, our ontology remains in its initial iteration. There is currently no systematic approach to quality assessment and verification. We will continue to expand and refine the ontology data. In the future, other dimensions and modalities should be added to the features, including EEG and imaging, to further improve the accuracy of classification and the completion of more downstream tasks.

### Conclusions

Clinically significant seizure information was successfully extracted from Chinese medical histories using NLP. This innovative approach represents a powerful tool for clinical research, with numerous potential applications, particularly for disorders characterized by complex clinical symptoms, such as seizure disorders. During this process, we constructed a bilingual ontology of seizure symptomatology comprising 702 terms. Furthermore, leveraging the extracted symptomatology information, we trained a binary classification model for generalized versus focal epilepsy using the stacking ensemble learning method. This demonstrates the feasibility of performing downstream tasks, such as seizure classification, based on the extracted information.

## Supplementary material

10.2196/57727 Multimedia Appendix 1 Supplementary materials.

10.2196/57727 Multimedia Appendix 2 Construction process of epilepsy semiology ontology.

10.2196/57727 Multimedia Appendix 3 Table S1. Purpose, scope, language and users of WWECA.

10.2196/57727 Multimedia Appendix 4 Model performance with different feature selection methods and sample ratios.

## Abbreviations

AUC: area under the curve
BFO: basic formalized ontology
DALYs: disability-adjusted life years
EEG: electroencephalogram
EHR: electronic health record
EPSO: epilepsy and seizure ontology
ESO: epilepsy semiology ontology
*ICD-10*: *International Classification of Diseases, Tenth Revision*
NLP: natural language processing
OWL: Ontology Web Language
ROC: receiver operating characteristic curve
SNOMED CT: Systemized Nomenclature of Medicine Clinical Terms
