# Supplementary material for: Semiology Extraction and Machine Learning–Based Classification of Electronic Health Records for Patients With Epilepsy: Retrospective Analysis
Source: JMIR Med Inform. 2024 Oct 17;12:e57727. doi: 10.2196/57727 (PMC11501417; doi:10.2196/57727)

**Decision Tree**

Parameters：

max_depth=10,

min_samples_leaf=3,

min_samples_split=18

**Random Forest**

Parameters：

n_estimators=170,

min_samples_split=2,

min_samples_leaf=2,

max_depth=14

**XGBoost**

Parameters：

learning_rate=0.02,

n_estimators=107,

max_depth=6,

min_child_weight=1,

subsample=0.5,

colsample_bytree=0.8,

gamma=0.4,

reg_alpha=0.3,

reg_lambda=0.6

**LightGBM**

Parameters：

learning_rate= 0.06,

n_estimators=121,

max_depth=14,

num_leaves=108,

subsample=0.5,

colsample_bytree=0.5,

reg_alpha=0.2,

reg_lambda=1.1,

min_child_samples=77


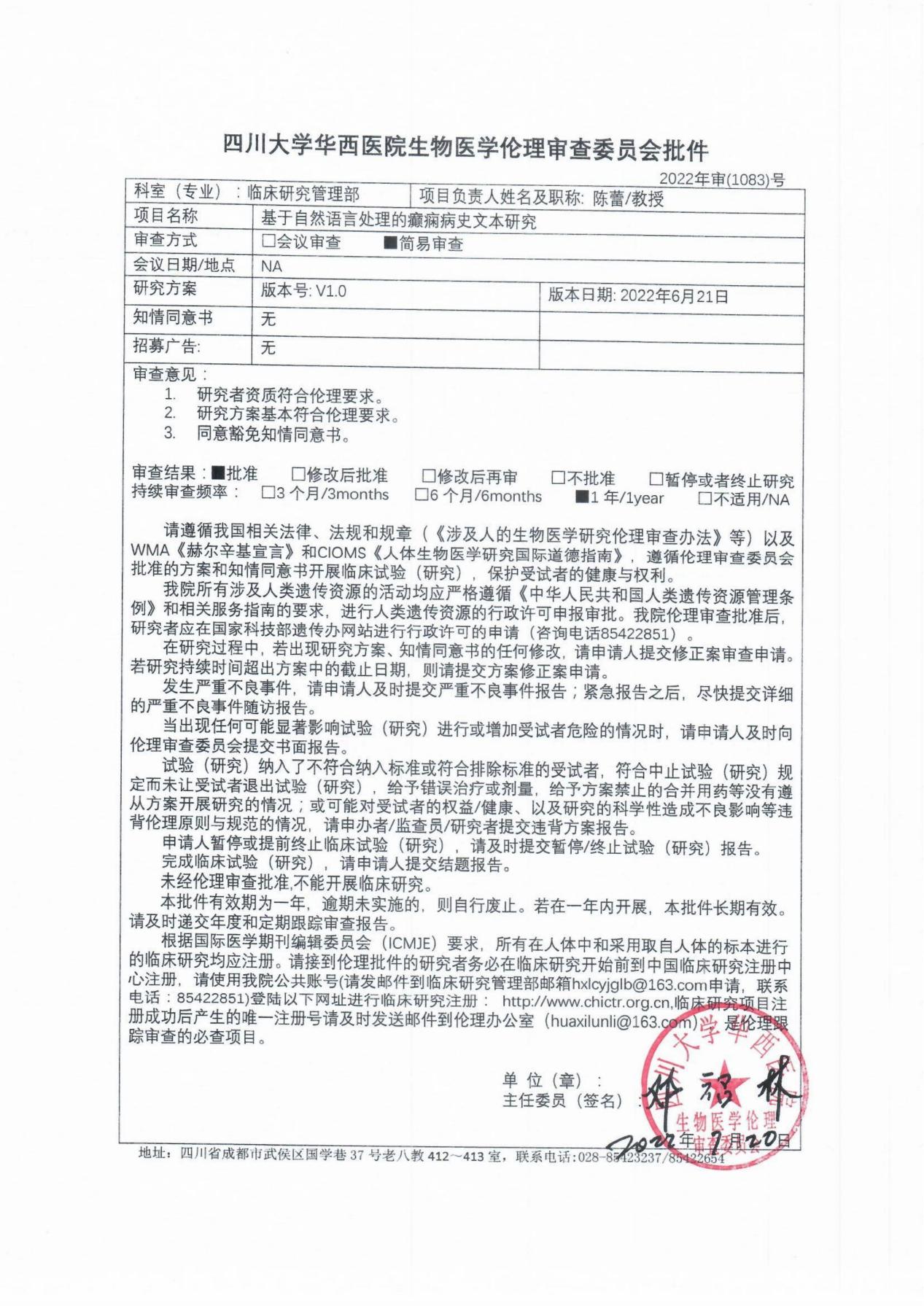


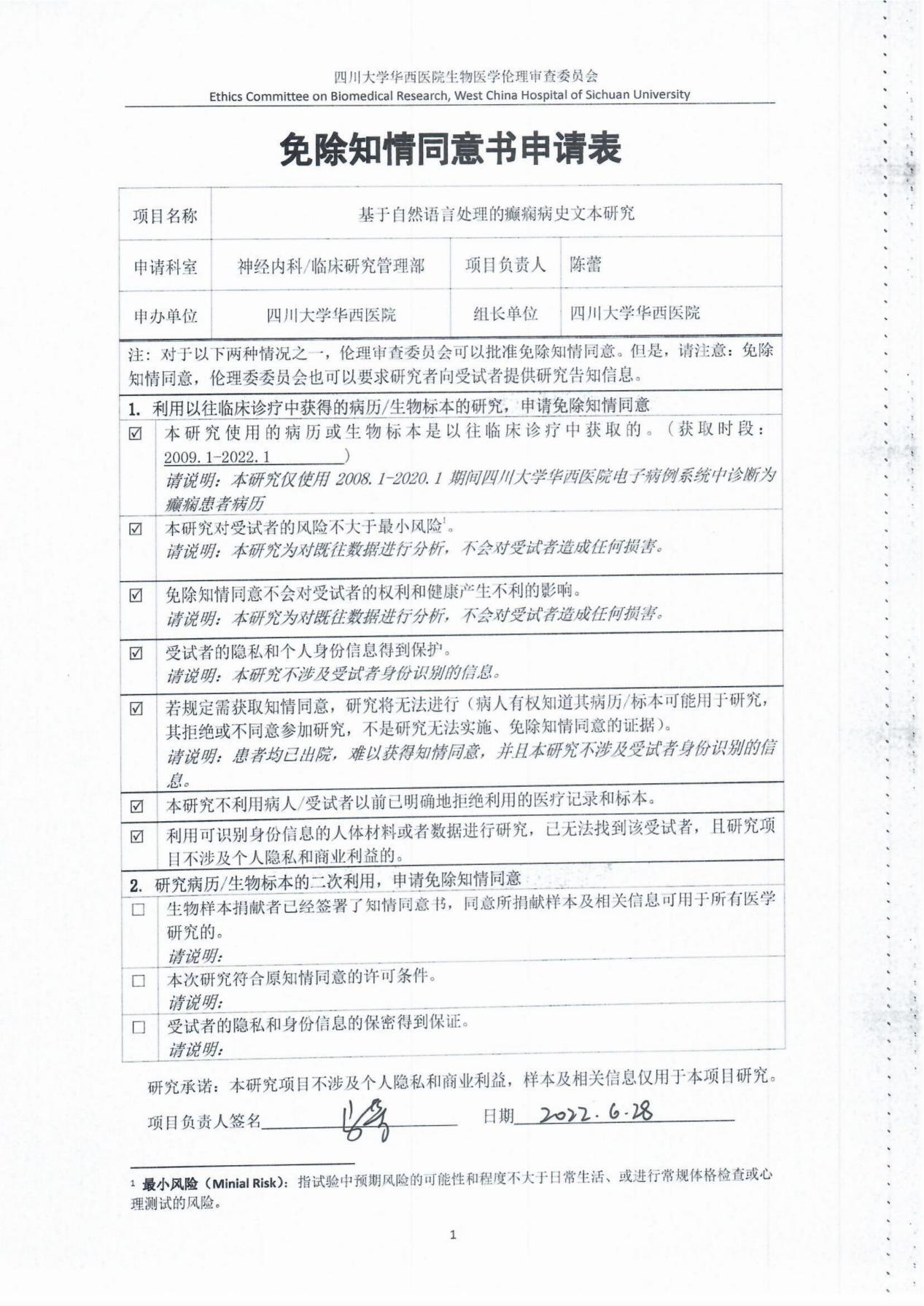

Supplement: Multimedia Appendix 1 [file medinform-v12-e57727-s001.docx]
