# Supplementary figures and images for: Semiology Extraction and Machine Learning–Based Classification of Electronic Health Records for Patients With Epilepsy: Retrospective Analysis
Source: JMIR Med Inform. 2024 Oct 17;12:e57727. doi: 10.2196/57727 (PMC11501417; doi:10.2196/57727)

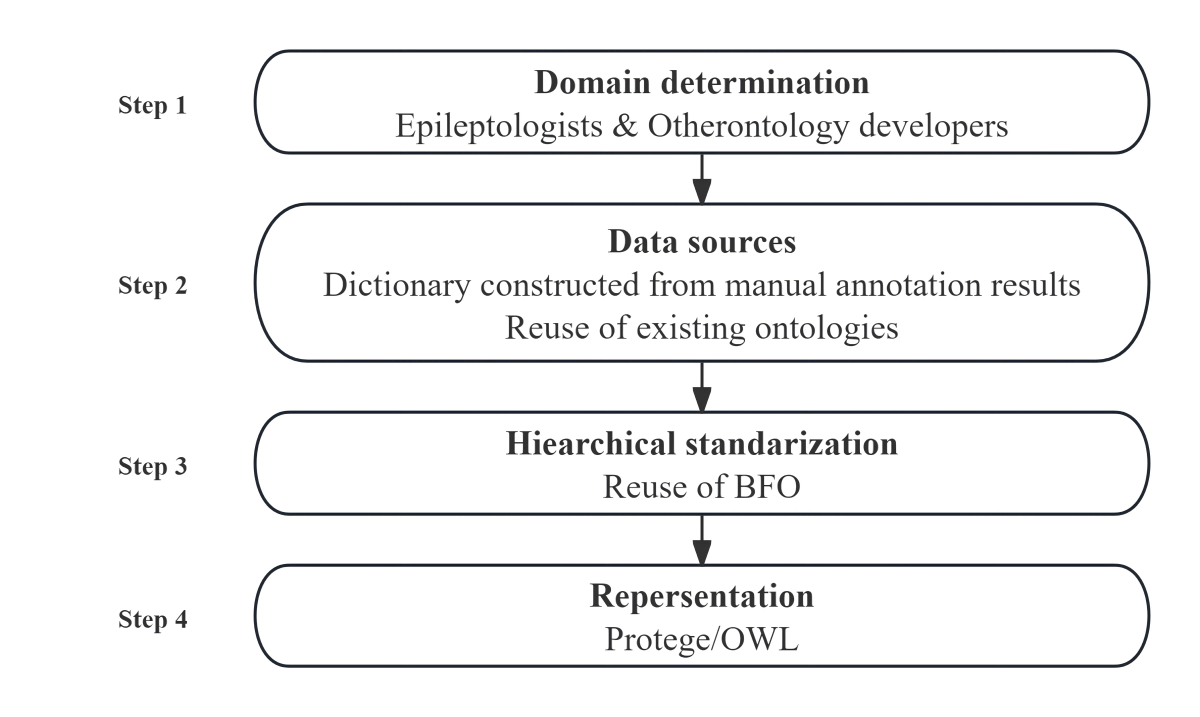

Supplement: Multimedia Appendix 2 [file medinform-v12-e57727-s002.png]

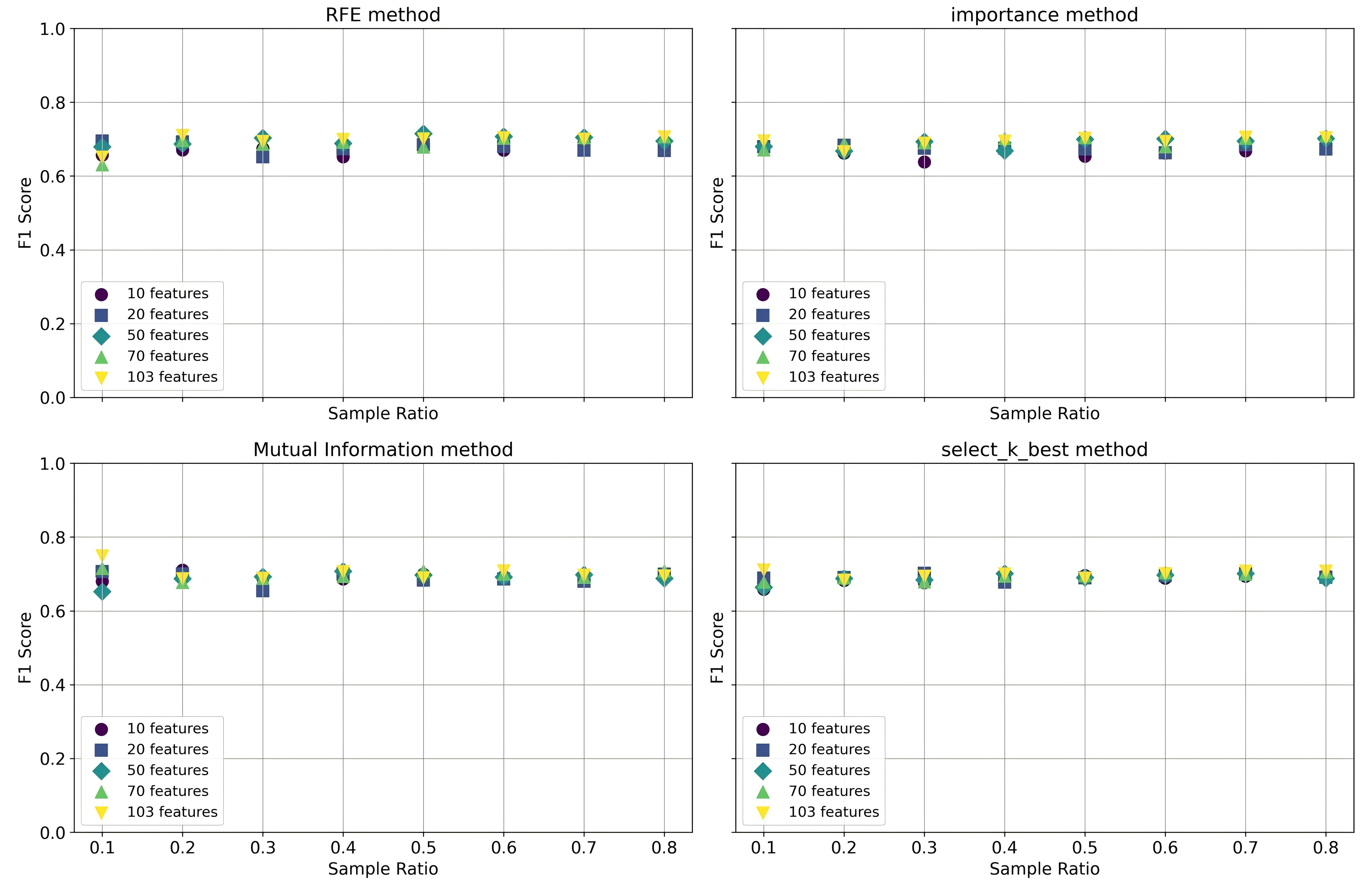

Supplement: Multimedia Appendix 4 [file medinform-v12-e57727-s004.png]
